# Induced Human-like Coronary Stenosis in Hypercholesterolemic PCSK9 Minipigs

**DOI:** 10.1007/s12265-025-10607-0

**Published:** 2025-03-25

**Authors:** Jacob Nicolaisen, Christian Frøsig Bo Poulsen, Martin Mæng Bjørklund, Martin Nors Skov, Maiken Kudahl Larsen, Troels Thim, Jouke Dijkstra, Jacob Fog Bentzon, Evald Høj Christiansen, Niels Ramsing Holm

**Affiliations:** 1 Department of Cardiology, Aarhus University Hospital, Aarhus, Denmark; 2 Institute of Clinical Medicine, Aarhus University, Aarhus, Denmark; 3 Department of Anesthesiology and Intensive Care, University Hospital of Southwest Jutland Esbjerg, Esbjerg, Denmark; 4 Department of Paediatrics and Adolescent Medicine, Lillebaelt Hospital, University Hospital of Southern Denmark, Kolding, Denmark; 5 Department of Regional Health Research, University of Southern Denmark, Odense, Denmark; 6 Department of Forensic Medicine, Aarhus University Hospital, Aarhus, Denmark; 7 Division of Image Processing, Leiden University Medical Center, Leiden, Netherlands

**Keywords:** Atherosclerosis, Animal model, Fibroatheroma, Percutaneous coronary intervention, Obstructive coronary stenosis, Bioresorbable stent

## Abstract

Translational models for obstructive coronary artery disease are lacking. We aimed to develop a porcine model for obstructive coronary stenosis induced by bioresorbable stent (BRS) implantation in hypercholesterolemic proprotein convertase subtilisin/kexin-9 (PCSK9) minipigs. Fifteen hypercholesterolemic PCSK9 minipigs were randomized to percutaneous coronary intervention with Magmaris, Absorb or Desolve BRS. Optical coherence tomography (OCT) scans were performed at baseline and 6-months followed by histology. Matched OCT analysis showed minimal lumen area decreased from 7.62 ± 1.54 mm^2^ at baseline to 2.12 ± 0.92 mm^2^ at follow-up in the Magmaris group, from 6.99 ± 1.49 mm^2^ to 3.07 ± 1.52 mm^2^ in the Absorb group, and from 6.05 ± 1.11 mm^2^ to 2.65 ± 0.93 mm^2^ in the Desolve group. Histologic examination revealed advanced human-like stenosis. BRS implantation in hypercholesterolemic PCSK9 minipigs induced human-like stenoses and may serve as a feasible preclinical model for obstructive coronary artery disease.

**Graphical Abstract:**

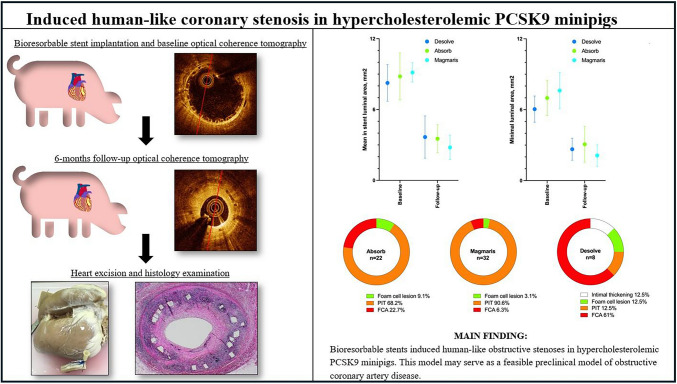

**Supplementary Information:**

The online version contains supplementary material available at 10.1007/s12265-025-10607-0.

## Introduction

Coronary artery disease (CAD) is a leading cause of death and disability worldwide [1]. Symptomatic CAD is often caused by obstructive lesions and affected patients are routinely treated both medically and with percutaneous coronary intervention (PCI) by implantation of one or more permanent metallic drug-eluting stents (DES) [2].

Despite advances in treatment, there is a substantial unmet need for disease models for preclinical evaluation of new treatments and optimization of existing strategies. Studying CAD is challenging in a preclinical setting since the coronary arteries are not amenable to investigation in mouse or rabbit models. Porcine models are more appropriate owing to the anatomic similarities, but published transgenic models and pigs on high cholesterol diets have only shown very limited potential for development of obstructive CAD [3–10].

The transgenic Yucatan hypercholesterolemic minipig overexpressing proprotein convertase subtilisin/kexin 9 (PCSK9) consistently develops human-like atherosclerosis when placed on a high fat high cholesterol diet. The PCSK9 pigs show advanced plaque types such as fibrous cap atheromas in the coronary arteries but rarely develops human-like obstructive stenosis [10]. Implantation of BRS have shown a markedly neointima response in pigs [11] and we hypothesized that the BRS would induce neoatherosclerosis resembling human obstructive stenosis in the PCSK9 minipig.

Thus, the aim of the present study was to assess the development of human-like obstructive coronary stenosis following BRS implantation in a porcine model prone to development of advanced atherosclerotic lesions.

## Methods

The present study is a prospective, randomized, proof-of-concept animal study assessing the development of stenosis and neo-atherosclerosis following BRS implantation in transgenic PCSK9 minipigs. The study was approved by the national institution The Animal Experiments Inspectorate, Glostrup, Denmark. The study was performed in accordance with relevant guidelines and regulations, and in compliance with European legislation (Directive 2010/63/EU). The study is reported as per the ARRIVE guidelines [12].

### The Hypercholesterolemic PCSK9 Porcine Model

The D374Y-PCSK9 Yucatan minipig overexpresses a gain of function human PCSK9 in hepatocytes causing degradation of hepatic LDL receptors and leading to hypercholesterolemia and atherosclerosis when fed a high fat high cholesterol (HFHC) diet [10]. A total of 15 PCSK9 minipigs were fed a cholate free, HFHC diet for at least one month prior to PCI treatment and continued on the diet until completion of the study. The diet and feeding regimen used have been described previously [6, 10].

### Implanted Devices

Three CE-marked BRS were evaluated. The magnesium based Magmaris BRS (Biotronik, Germany) with a degradation time of 6–12 months [13]. The poly-L lactic acid (PLLA) based Absorb BRS (Abbott, USA) degrading in 3–4 years [14], and the Desolve PLLA BRS (Elixir, USA) with limited self-expanding properties and a degradation time of 2–4 years [15].

### Experimental Protocol

The pigs were anesthetized and a 6F arterial sheath (Radifocus, Terumo, Tokyo, Japan) was placed percutaneously under ultrasound guidance. The coronary arteries were subsequently engaged using a 6 Fr AR1 guide catheter (Medtronic, Minneapolis, MN, USA) and the pigs were randomized by simple blinded draw to implantation with either Magmaris, Absorb, or Desolve BRS. The BRS were implanted in one or two coronary arteries (Left anterior descending artery, left circumflex artery or right coronary artery) at the operator’s discretion. In case of clinical unstable conditions or unstable guiding catheter position, only one vessel was implanted with a BRS. The size of the BRS was intended to be 0.5 mm smaller than the reference size based on OCT to achieve slight under-expansion of the BRS. OCT scans were performed pre- and post-stenting. In case of severe malapposition detected by OCT, post-dilatation was performed followed by a final OCT acquisition. Further details regarding the experimental protocol are outlined in the supplemental methods.

Planned follow-up was performed 6 months later. Three pigs had an additional follow-up at one year.

### Medication

One week prior to implantation of the BRS, all pigs received 300 mg aspirin (Takeda Pharma, Roskilde, Denmark) daily. Within 24 h of the procedure, 300 mg of clopidogrel (Actavis Group, Hafnarfjördur, Iceland) was administered. After BRS implantation, all pigs received 75 mg of aspirin and 75 mg of clopidogrel once daily for the remainder of the study. Unfractionated heparin 5000U (LEO Pharma, Ballerup, Denmark) was administered intravenously and supplemented with a further 1000u each hour during BRS implantation and during follow-up procedures.

### Optical Coherence Tomography Acquisition and Analysis

Optical coherence tomography scans were performed with either optical frequency domain imaging (OFDI, Terumo Medical Corporation, Japan) or the Ilumien OCT system (Abbott Vascular, USA). The OCT recordings were analyzed in QCU-CMS (Leiden University, Netherlands). Baseline and follow-up OCT scans were matched on frame level by identifiable landmarks including side branches, stent edges and calcified plaques. OCT acquisitions were analyzed with a sample rate of 1 mm.

The adjustable strut analysis in QCU-CMS was used for the Desolve and Absorb BRS. The reflective Magmaris stent struts inhibited visualization of the abluminal boarder, hence analysis with fixed strut thickness was utilized in the Magmaris group. Detailed description of the OCT analysis is outlined in the supplemental methods and Supplementary Fig. 1.

Lesions were characterized according to the following definitions for obstructive disease: diameter stenosis ≥ 50% or ≥ 70%, area stenosis ≥ 50% or ≥ 70%, minimal lumen area ≤ 2.5 mm.

### Tissue Preparation and Histology

After the final catheterization procedure, a median sternotomy was performed under general anesthesia and the heart exposed. Unfractionated heparin (10,000u) was injected intravenously followed by injection of 50 mL saturated potassium chloride solution (KPBS) into the ascending aorta under direct vision. The heart was excised, and the coronary arteries flushed with KPBS. Next, the coronary arteries were perfusion-fixed at 100 mmHg for 1 h, followed by immersion fixation for 24 h in 4% formaldehyde diluted in PBS (VWR, Prolabo, Belgium), after which the heart was stored in PBS at 5 °C until further processing.

The coronary arteries with the most severe stenosis as determined by in vivo OCT for each of the three stent types were analyzed by histology. The selected stented segments were paraffin embedded, processed, and sectioned each 1.0 mm followed by staining with haematoxylin & eosin and with Sirius red. The microscopic examination was performed for each 1.0 mm using an Olympus Bx50 light microscope. The in-stent coronary lesions were qualitatively assessed by two expert pathologists blinded to the OCT scans. Lesions were classified according to Virmani [16]. In case of discrepancy in plaque classification, a consensus between the two pathologists were achieved. 

### Statistics

Categorical variables are reported as counts and percentages. Continuous variables are reported as mean with standard deviations if following a gaussian distribution or as medians with interquartile range if not. Comparison of matched baseline and follow-up results from the three BRS groups were performed by one-way ANOVA if following a gaussian distribution or else by Kruskal–Wallis test. Comparison of histology findings from the three BRS groups were performed by Fischer’s exact test. Normal distribution was assessed with QQ-plots and histograms. Bartlett’s test was used to test for equal variances. In case of unequal variances, the Kruskal–Wallis test was used. Each coronary vessel implanted with BRS was considered an independent observation.

## Results

### Study Pigs

**Fig. 1:**
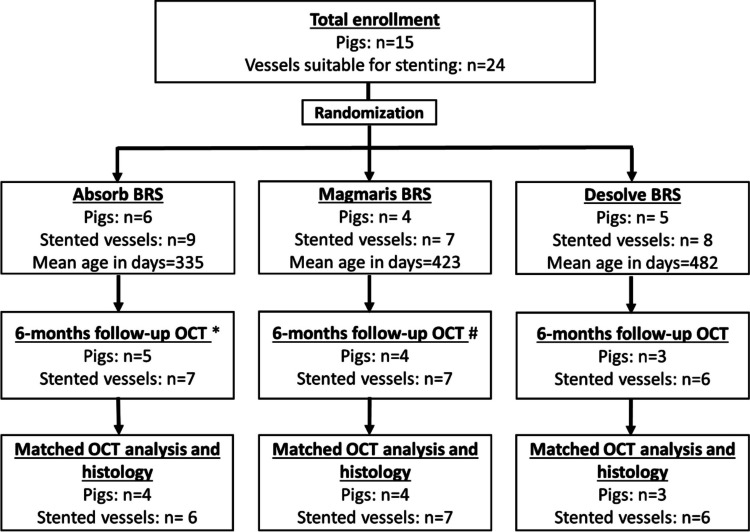
Flowchart. Figure 1 Outline of the study showing number of pigs, stented vessels, and the mean age at baseline procedure in the three BRS groups. Three pigs died during follow-up (one in the Absorb group, two in the Desolve group. An additional two pigs from the Desolve group died at final follow-up procedure. The OCT-recordings from those pigs were acquired immediately post-mortem. One Absorb pig (*n* = 1 stented vessel) was excluded from matched OCT analysis due to wobbly wire artefact. * One pig (*n* = 1 stented vessel) had OCT follow-up after 4.5 months. Another pig (*n* = 1 stented vessels) had OCT follow-up after 3.5 months. #: One pig (*n* = 1 stented vessel) had OCT follow-up at 3.5 months

A timeline of events is presented in Fig. 1. Of the 15 PCSK9 minipigs included in the study, three pigs died before follow-up. This included two pigs implanted with Desolve BRS; one died due to access-site bleeding, whereas autopsy did not reveal the cause of death in the other pig but ruled out access-site bleeding and stent thrombosis. A pig implanted with Absorb died without signs of access site bleeding, but an autopsy was not performed. A total of 12 pigs (*n* = 20 stented vessels) had OCT evaluation at 6-months. One scan was subject to severe artefacts leaving 19 stented vessels in the matched OCT analysis. Nine pigs were sacrificed following the OCT evaluation at 6-month. Three pigs had an additional OCT follow-up at 12-month post-implantation before being sacrificed. In total, two Magmaris implanted vessels were sectioned into 32 sections, two Absorb implanted vessels into 22 sections, and one Desolve implanted artery into 8 sections.

Baseline and follow-up characteristics are presented in Table 1 and Supplementary Table 1.

**Table 1:** Baseline and follow-up characteristics

PCSK9 minipigs (*n* = 11)	Magmaris (*n* = 4)	Absorb (*n* = 4)	Desolve (*n* = 3)
Age at procedure (days)	423 ± 178 (175–553)	335 ± 180 (175–552)	482 ± 70 (412–552)
Weight (kg) Baseline Follow-up	83 ± 31 (42–109) 96 ± 26 (62–117)	63 ± 34 (29–107) 79 ± 23 (51–106)	95 ± 19 (73–106) 108 ± 14 (92–118)
Cholesterol (mmol/L) Baseline Follow-up	27.4 ± 6.0 (21.6–35.8) 21.0 ± 3.62 (17.9–26.1) *	26.3 ± 4.7 (21.9–31.5) 20.7 ± 2.1 (18.9–23.4) †	19.1 ± 3.65 (16.2–23.2) 20.9 ± 5.52 (14.5–24.4)

Results are presented as mean ± SD (minimum–maximum). *1 pig with follow-up cholesterol measurement at 1-year follow-up. † 2 pigs with follow-up cholesterol measurement at 1-year follow-up

### Qualitative OCT Results

**Fig. 2:**
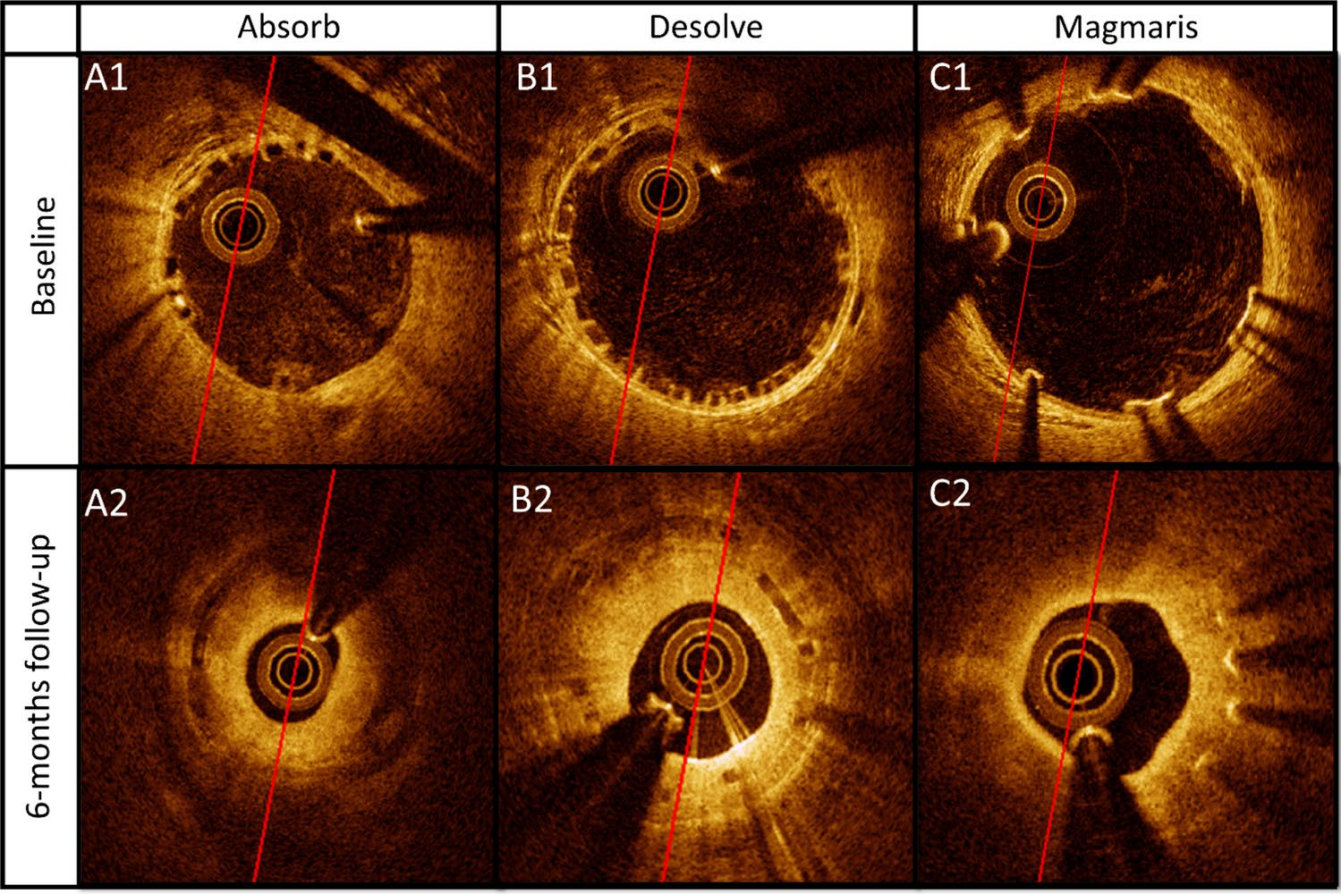
Matched baseline and 6-months follow-up optical coherence tomography. Figure 2 Segments with minimal lumen area at 6-months follow-up (**A2**, **B2**, **C2**) and corresponding post-stenting baseline image (**A1**, **B1**, **C1**) for Absorb BRS, Desolve BRS, and Magmaris BRS respectively. #, part of the pericardial space with a small amount of fluid is shown in the top of the image

**Fig. 3:**
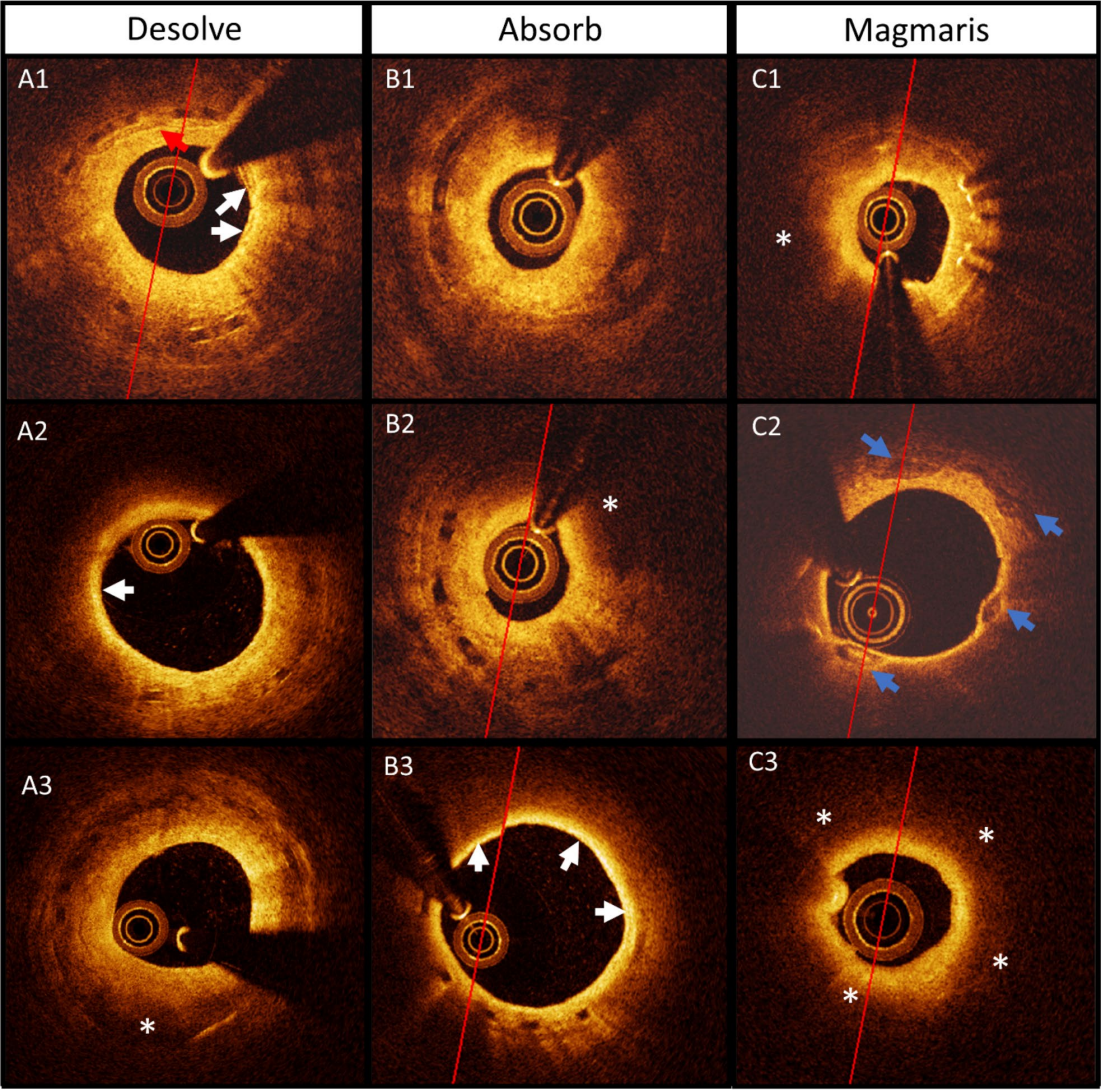
Lesion assessment by optical coherence tomography. Figure 3 In-stent coronary artery lesions 6 months after BRS implantation, depicted by OCT. **A1**: Lesion in Desolve BRS showing cholesterol crystals (red arrows) and area with highly reflective tissue with signal attenuation indicative of inflammation and foamy-macrophage accumulation (white arrows). **A2**: Lesion in Desolve BRS with inflammation (white arrow). **A3**: Lesion in Desolve BRS with areas of signal attenuation that could indicate lipid accumulation (white asterisk). **B1** Neoatherosclerotic stenosis in Absorb BRS with a minimal lumen area of 0.94 $m{m}^{2}$ at 6-months follow-up. **B2**: Lesion in Absorb BRS showing areas with possible lipid accumulation (white asterisk). **B3**: Lesion in Absorb BRS with inflammation or possibly macrophages (white arrows). **C1** + **C3**: Lesion in Magmaris BRS showing areas with possibly lipid accumulation (white asterisk). **C2**: Lesion in Magmaris BRS at 12-months follow-up showing areas with calcification (blue arrows)

**Fig. 4:**
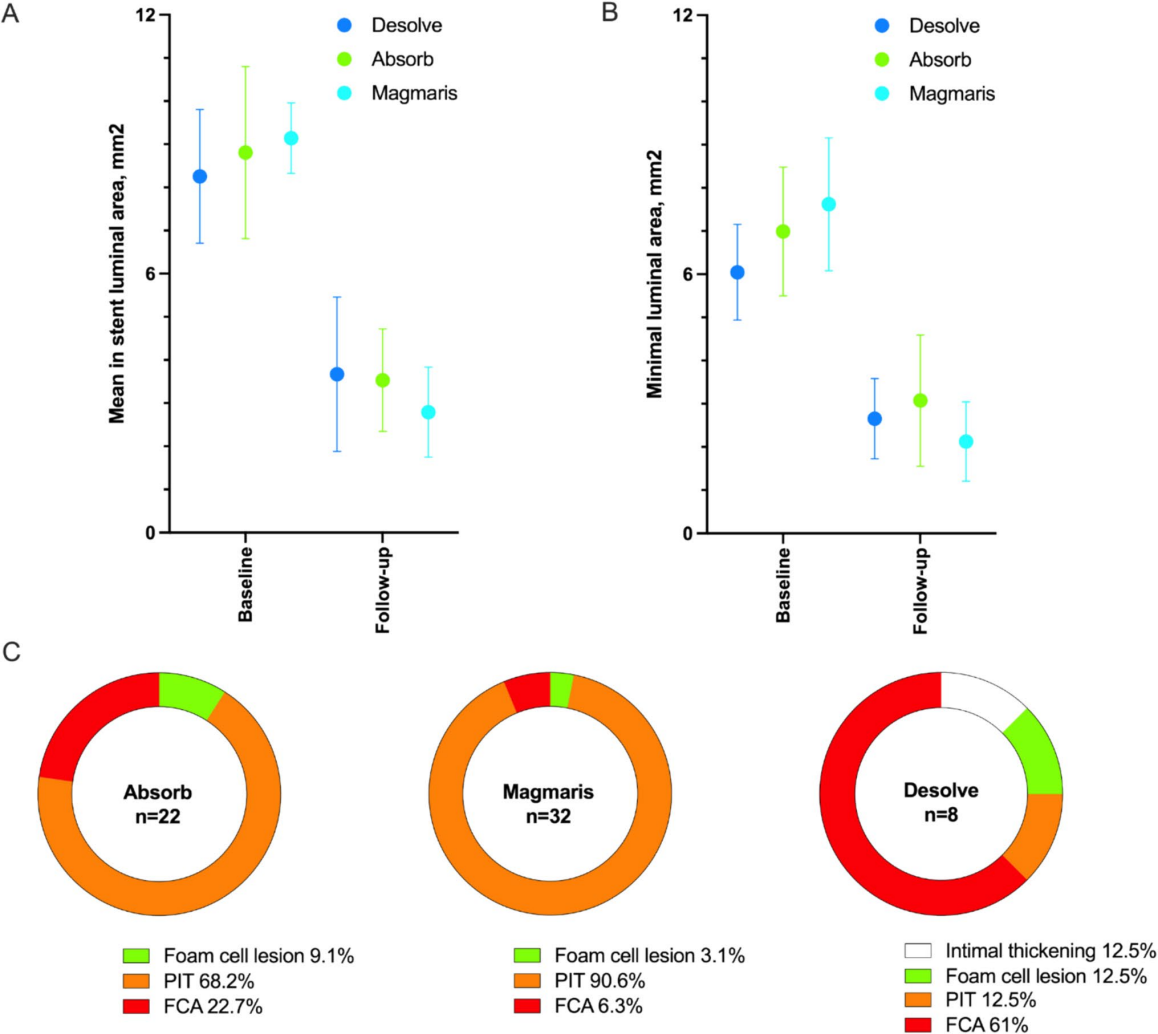
Luminal area and lesion classification. Figure 4 Change in mean luminal area (**A**) and minimal luminal area (**B**) from baseline to 6-months follow-up by OCT. **C**: Distribution of lesion types in Absorb (*n* = 22 sections), Magmaris (*n* = 32 sections) and Desolve (*n* = 8 sections) BRS implantations. PIT = Pathological intimal thickening. FCA = Fibrous cap atheroma

**Fig. 5:**
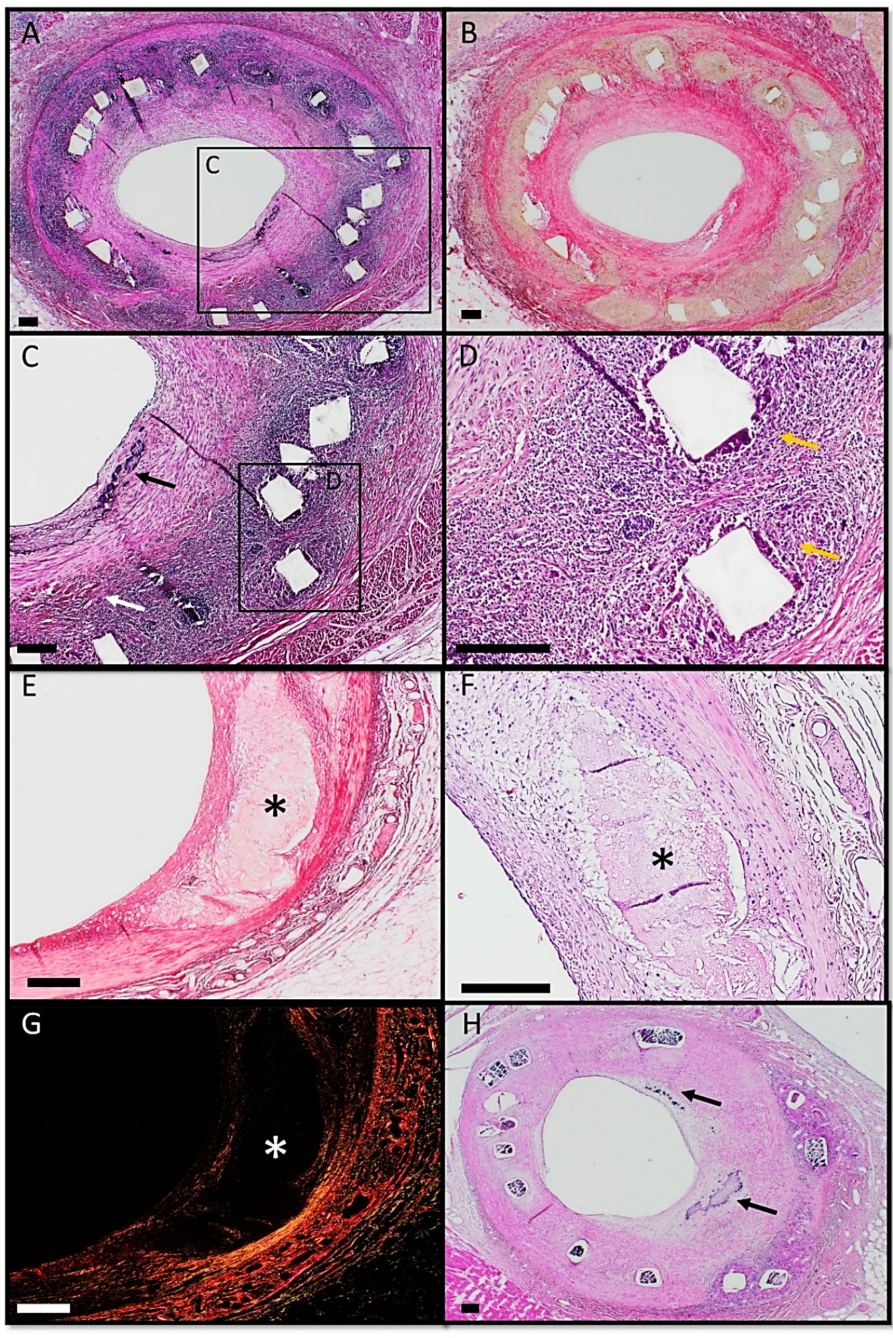
Induced coronary stenosis evaluated by histology. Figure 5 Series of histology sections of vessels implanted with Desolve BRS (A + B + C + D), Absorb BRS (E + F + G) and Magmaris BRS (H). Scale bar indicates 150 µm. **A**: H&E stain showing inflamed neointima with peri-strut infiltration of inflammatory cells. **B**: Sirius-stain of B. C: Higher magnification view of peri-strut inflammation, neoangiogenesis (white arrow) and micro calcification (black arrow). **D**: Higher magnification view showing peri-strut infiltration of inflammatory cells indicating inflammation (yellow arrows). **E**: Sirius stained fibroatheroma with necrotic core formation (aterisk) in an Absorb implanted vessel 12 months post-PCI. **F**: H&E stained fibroatheroma with a fibrous cap and a necrotic core (Asterisk). **G**: Sirius stained fibroatheroma under polarized light showing lack of collagen in the necrotic core (Asterisk) beneath a fibrous cap. H: H&E-stained fibroatheroma and calcification (black arrows)

Coronary stenoses were observed at follow-up after implantation with all of the three BRS (Figs. 2 and 3). The stenoses showed advanced neo-atherosclerosis including fibroatheroma, pathological intimal thickening, accumulation of inflammatory cells, neointimal microvessels and calcifications. The histologic examination and plaque distribution can be appreciated in Figs. 4 and 5.

**Fig. 6:**
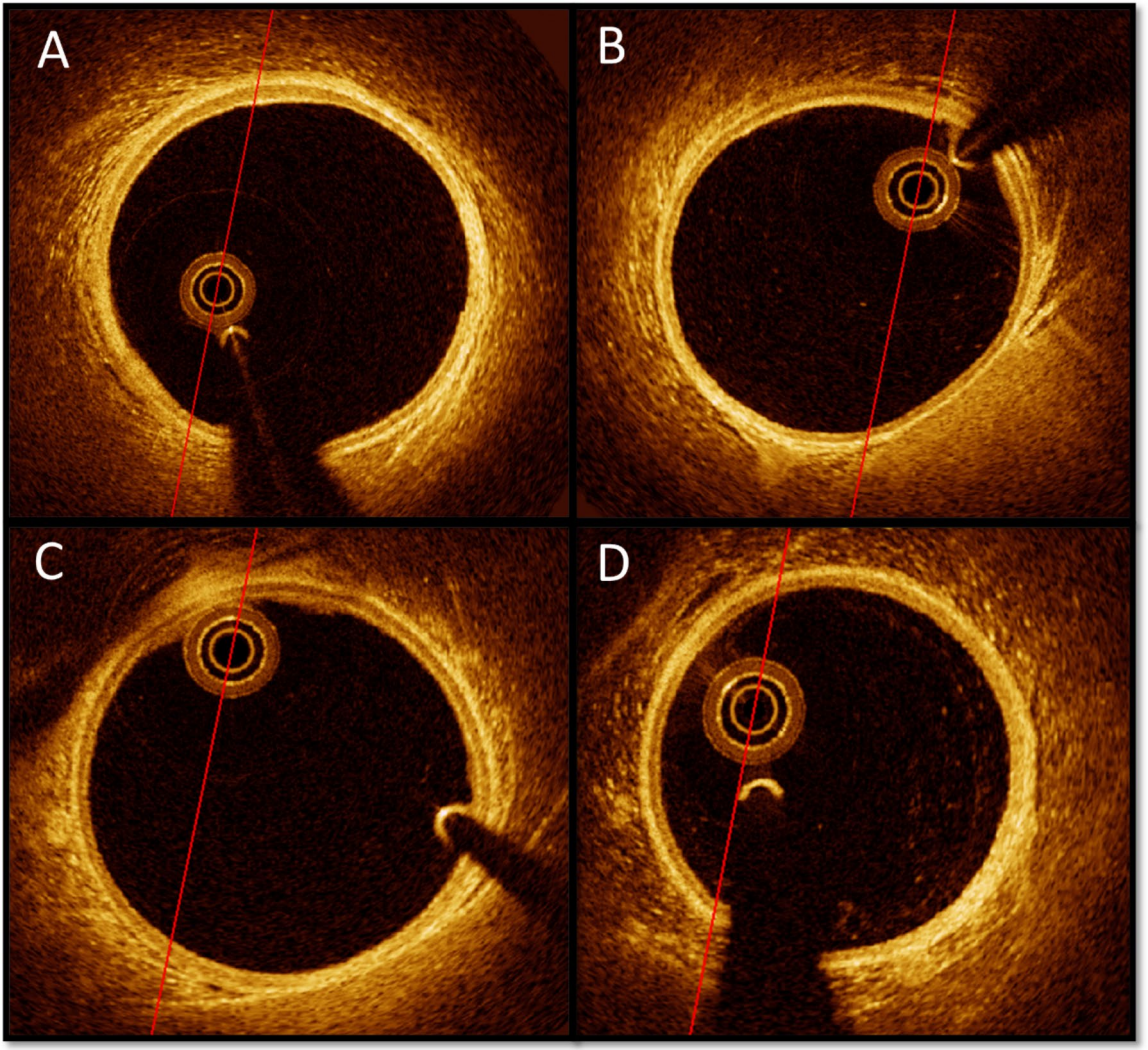
Optical coherence tomography findings in non-stented segments at 6-months follow-up. Figure 6 Six months follow-up cross-sectional OCT-images 5–15 mm proximal to the proximal stent edge. **A**: Right coronary artery. **B**: Left circumflex artery. **C**: Left anterior descending artery. **D**: Left anterior descending artery

The coronary arteries appeared normal and healthy 5–15 mm proximal to the stented segments No obstructive coronary artery disease was observed in the non-stented segments used as reference (Fig. 6).

### Quantitative OCT Results

The matched OCT results are presented in Table 2, Table 3 and Supplementary Table 2. The minimal lumen area (MLA), decreased from 7.62 ± 1.54 ${\text{mm}}^{2}$ at baseline to 2.12 ± 0.92 ${\text{mm}}^{2}$ at follow-in the Magmaris group, from 6.99 ± 1.49 ${\text{mm}}^{2}$ to 3.07 ± 1.52 ${\text{mm}}^{2}$ in the Absorb group and from 6.05 ± 1.11 ${\text{mm}}^{2}$ to 2.65 ± 0.93 ${\text{mm}}^{2}$ in the Desolve group (Fig. 4 and supplementary Fig. 2). Differences in MLA at 6-months between BRS groups were non-significant (p = 0.35).

**Table 2:** Matched baseline and 6-months follow-up OCT

OCT endpoints (*n* = 19 stented vessels)	Magmaris (*n* = 7)	Absorb (*n* = 6)	Desolve (*n* = 6)	P-value
Minimal lumen area (${\varvec{m}}{{\varvec{m}}}^{2}$)				
Baseline: Follow-up: Loss in minimal lumen area:	7.62 ± 1.54 2.12 ± 0.92 5.50 ± 2.29	6.99 ± 1.49 3.07 ± 1.52 3.92 ± 1.28	6.05 ± 1.11 2.65 ± 0.93 3.40 ± 1.65	0.16 0.35 0.12
Mean lumen area (${\varvec{m}}{{\varvec{m}}}^{2}$)				
Baseline: Follow-up: Loss in mean lumen area:	9.14 ± 0.82 2.79 ± 1.04 6.35 ± 1.53	8.81 ± 1.99 3.82 ± 1.31 4.99 ± 1.63	8.26 ± 1.55 3.67 ± 1.79 4.58 ± 2.99	0.58 0.37 0.31
Mean stent area (${\varvec{m}}{{\varvec{m}}}^{2})$				
Baseline: Follow-up: Loss in mean stent area:	9.72 ± 0.85 5.92 ± 1.09 3.80 ± 1.65	8.86 ± 2.11 7.77 ± 1.59 1.10 ± 0.96	8.17 ± 1.48 8.86 ± 3.72 −0.69 ± 4.91	0.22 0.09 0.03
Minimal stent area (${\varvec{m}}{{\varvec{m}}}^{2})$				
Baseline: Follow-up: Loss in minimal stent area:	8.48 ± 1.51 4.89 ± 0.89 3.59 ± 2.26	7.26 ± 1.73 6.61 ± 1.71 0.65 ± 1.14	6.15 ± 1.17 7.02 ± 2.21 −0.87 ± 3.07	0.04 0.07 0.009
Mean neointimal hyperplasia area ($\mathbf{m}{\mathbf{m}}^{2})$				
Baseline: Follow-up:	- 2.94 ± 0.43	- 3.67 ± 0.85	- 4.90 ± 2.31	- 0.12
Strut malapposition (%) * †				
Baseline: Follow-up:	0 [0.0;0.0] 0 [0.0;0.0]	3.7 [0.0;28] 0 [0.0;0.0]	11.1 [0.0;18.4] 0 [0.0;0.0]	0.11 -

Results are reported as mean ± SD. *Median [IQR]. †Analyzed on strut level

**Table 3:** Obstructive lesions after 6 months by OCT

OCT evaluation (*n* = 19 stented vessels)	Magmaris (*n* = 7)	Absorb (*n* = 6)	Desolve (*n* = 6)	P-value
Area stenosis ≥ 50%	7 (100%)	6 (100%)	5 (83%)	0.632
Area stenosis ≥ 70%	5 (71%)	1 (17%)	1 (17%)	0.109
Diameter stenosis ≥ 50%	5 (71%)	1 (17%)	1 (17%)	0.109
Diameter stenosis ≥ 70%	0 (0%)	0 (0%)	0 (0%)	-
Lumen area ≤ 2.5mm^2^	5 (71%)	3 (50%)	3 (50%)	0.722

Results are reported as n (%)

The largest loss in mean stent area was found in the Magmaris group from 9.72 ± 0.85 ${\text{mm}}^{2}$ to 5.92 ± 1.09 ${\text{mm}}^{2}$, compared with 8.86 ± 2.11 ${\text{mm}}^{2}$ to 7.77 ± 1.59 ${\text{mm}}^{2}$ in the Absorb group whereas mean stent area increased from 8.17 ± 1.48 ${\text{mm}}^{2}$ to 8.86 ± 3.72 ${\text{mm}}^{2}$ after implantation of the Desolve BRS with self-expanding properties (Supplementary Fig. 3). Loss in minimal stent area differed significantly between groups (p = 0.009, Table 2).

The classification of lesions according to five definitions for obstructive stenosis are shown in Table 3.

Stent strut malapposition at baseline was 0% [0.0;0.0] for Magmaris BRS, 3.7% [0;28] % for the Absorb BRS and 11.1% [0.0;18.4] for the Desolve BRS (*p* = 0.11). No malapposition was observed at follow-up in any implanted BRS. At 6-months follow-up the mean neointimal hyperplasia area was 2.94 ± 0.43 ${\text{mm}}^{2}$ in Magmaris BRS, 3.67 ± 0.85 ${\text{mm}}^{2}$ in Absorb BRS, and 4.90 ± 2.31 ${\text{mm}}^{2}$ for Desolve implantations (*p* = 0.12).

The three pigs with extended 12-months follow-up showed no additional loss of mean or minimal lumen area compared to 6-months follow-up (Supplementary Fig. 4).

### Histology

In-stent quantitative histology results are presented in Fig. 4. Frequency of fibroatheromas differed significantly between the three BRS groups; 6.3% in the Magmaris group, 22.7% in the Absorb group and 62.5% in the Desolve group (*p* = 0.002).

Qualitative histological examination revealed advanced plaque features (Fig. 5) including peri-strut inflammation, pathological intimal thickening, neointimal microvessels, calcifications, and fibroatheroma with necrotic core formation.

## Discussion

The main findings of this study on implantation of BRS for inducing coronary stenosis in hypercholesterolemic PCSK9 minipigs were: 1) severe in-scaffold stenosis developed in PCSK9 minipigs implanted with any of the three BRS types during 6 months of follow-up. 2) Serial evaluation of changes in lumen area from 6-months follow-up to 1-year follow-up indicated no additional benefit in terms of further loss in lumen area. 3) The morphology of the BRS-induced lesions showed advanced, human-like neo-atherosclerosis, including neointimal microvessels, calcifications, infiltration of inflammatory cells and necrotic core formation.

Our findings indicate that PCSK9 minipigs implanted with BRS could emerge as a preclinical model for human-like obstructive coronary artery disease. Such a model may allow for investigation of disease processes and enable better pre-clinical evaluation of drugs, implanted devices, treatment techniques, diagnostic tests, and coronary imaging modalities.

### Comparison of Atherosclerotic Porcine Models

The level of hypercholesterolemia induced by the PCSK9 mutation was comparable to other studies of the PCSK9 minipig [6–10]. Previous studies reported development of coronary atherosclerosis, but more severe stenosis was only developed in very few animals if any [3–10]. Implantation of a permanent shear-modifying stent in PCSK9 minipigs led to development of advanced plaques including thin-cap-fibroatheroma [6], however the presence of a permanent foreign body like the shear-modifying stent may limit the applicability as an obstructive model due to altered mechanical properties and continuous mechanically induced inflammation. Another study evaluated balloon injury combined with an atherogenic diet in downsized Rapacz pigs with a mutated LDL-receptor to accelerate atherosclerosis. Advanced plaques were observed, however they were generally non-obstructive [4]. An Ossabaw porcine model with metabolic syndrome was shown to induce restenosis following permanent metallic DES implantations but the lesions were not obstructive [5]. Another Ossabaw pig model expressing the D374Y-PCSK9 mutation also developed plaques with only mild stenosis [3]. In the present study, the human-like obstructive stenosis was consistently developed in implanted BRS. With BRS’ potential for inducing anatomic significant stenoses and simultaneous resorption, the model may enable studying natural history treatments of obstructive CAD and thereby potentially overcoming the limitations of present preclinical models.

### Response According to BRS type

The substantial decrease in minimal lumen area (MLA) observed at follow-up did not differ significantly between the three BRS groups, but the loss in MLA was numerically greater after Magmaris BRS implantations. A finding that could be explained by the rapid resorption process of this device, with complete absorption occurring in less than a year [17].

Thus, Magmaris BRS could be particularly suitable for this animal model as the time from implantation to full BRS resorption may be an important factor in emulation of a native human coronary stenosis. The Absorb stent area remained stable throughout follow-up whereas stent area of the Desolve BRS showed a slight increase, most likely due to two implantations from the same pig that showed a large positive vessel remodellering. The findings may be consistent with the Desolve BRS’ proven self-expanding capacity [18].

### Coronary Artery Lesion Morphology

Qualitative OCT assessment identified neo-atherosclerosis, neointimal micro-vessels, calcifications and lipid plaques in the induced lesions. Limitations of OCT could influence characterization of the model. Lipid-filled macrophages in the luminal part of the neointima can create signal attenuation inhibiting evaluation of structures underneath and tangential drop-out artefacts in some instances mimics a fibroatheroma [19]. Signal-poor areas indicating lipid plaques may also be created by fibrin accumulation, granulation tissue or large infiltrations of inflammatory cells [20].

To aid the characterization, the lesions were assessed by histologic examination that identified advanced neoatherosclerosis with human like features of calcifications, peri-strut inflammation, neoangiogenesis, and necrotic core formation. Fibroatheromas were observed in all three BRS groups. Importantly no atheromatosis were found in the non-stented vessels. Previous studies of the PCSK9 minipig have also shown development of advanced plaques [6–10] but in the context of mostly non-obstructive atherosclerosis including fatty streaks.

### Clinical Perspectives

The BRS-induced stenoses in the PCSK9 porcine model may serve as an effective model to investigate natural history of atherosclerosis, and for evaluation of new diagnostic methods, treatment techniques, devices and drugs. Despite improvements in prognosis with “optimal medical therapy”, newer generation DES treatment [20–22] and refined surgical revascularization technique, there is still a substantial need for improvement of diagnostics and treatment.

The present model may enable device testing in a clinically relevant setting of elevated cholesterol. Indeed, previous studies of the Absorb BRS showed an excellent safety profile in non-atherosclerotic pigs [23], but investigation in humans raised safety concerns including increased levels of myocardial infarction and stent-thrombosis [24], ultimately leading to market withdrawal. The PCSK9 minipig might serve as an effective preclinical model with better correspondence to the patients treated clinically.

## Limitations

The limited sample size in each BRS treated group with limited power must be considered. To account for this, each implantation was considered statistically independent. There is a risk of dependency which might have influenced the results. Still, the general finding of induced atherosclerosis was seen in all animals.

Furthermore, despite the genetic, physiological, and anatomical similarities between pigs and humans, differences in development of neo-atherosclerosis should be considered.

The matching of OCT and histology was susceptible to cardiac motion artifacts and low sampling density.

Hematoxylin & Eosin and Sirius red were used as staining in the histological examination. No additional staining with more specific markers were used.

## Conclusion

Implantation of BRS in transgenic hypercholesterolemic PCSK9 minipigs induced formation of obstructive coronary stenosis. Lesions had human-like morphology with advanced plaque features including calcifications, lipid plaques with necrotic core, and neointimal microvessels. The PCSK9 minipig implanted with BRS may prove a feasible preclinical model for obstructive coronary artery disease.

## Clinical Relevance

Obstructive stenosis induced in the PCSK9 minipig may serve as an important and feasible preclinical model for investigations of plaque progression, pharmaceuticals, revascularization techniques, diagnostic modalities, and implantable devices for use in obstructive coronary artery disease.

## Supplementary Information

Below is the link to the electronic supplementary material.

Supplementary file1 (DOCX 5008 KB)

## Data Availability

The datasets generated during and analyzed during the current study are available from the corresponding author on reasonable request.

## Abbreviations

BRS: Bioresorbable stent
OCT: Optical coherence tomography
PCSK9: Proprotein convertase subtilisin/kexin type 9
PCI: Percutaneous coronary intervention
CAD: Coronary artery disease
MLA: Minimal luminal area
DES: Metallic drug-eluting stent
HFHC: High fat high cholesterol
PLLA: Poly-L lactic acid
LAD: Left anterior descending artery
RCA: Right coronary artery
LCX: Left circumflex artery

## Declarations

### Study Registration

Clinical trial registration number: Not applicable.

The present study was approved by the Danish Animal Experiments Inspectorate (2015–15-0201–00570). All institutional and national guidelines for the care and use of laboratory animals were followed and approved by the appropriate institutional committees. No human studies were carried out by the authors for this article.

### Human Studies

No human studies were carried out by the authors for this article. All institutional and national guidelines for the care and use of laboratory animals were followed and approved by the appropriate institutional committees.
