# Supplementary material for: Induced Human-like Coronary Stenosis in Hypercholesterolemic PCSK9 Minipigs
Source: J Cardiovasc Transl Res. 2025 Mar 25;18(3):544–55. doi: 10.1007/s12265-025-10607-0 (PMC12209001; doi:10.1007/s12265-025-10607-0)
Supplement: Supplementary file 1 — Supplementary file1 (DOCX 5008 KB) [file 12265_2025_10607_MOESM1_ESM.docx]

**Induced human-like coronary stenosis in hypercholesterolemic PCSK9 minipigs**

**Short title:** Coronary stenosis in hypercholesterolemic pigs

Jacob Nicolaisen^1,2,3^ MD, Christian Frøsig Bo Poulsen^1,2,4^ MD, PhD, Martin Mæng Bjørklund^1,2^ MD, Martin Nors Skov^2^ MSc, Maiken Kudahl Larsen MD^5^, PhD, Troels Thim, MD, PhD^1,2^, Jouke Dijkstra^6^, PhD, Jacob Fog Bentzon^1,2^ MD, PhD, Evald Høj Christiansen^1,2^ MD, PhD, Niels Ramsing Holm^1,2^ MD

^1^Department of Cardiology, Aarhus University Hospital, Aarhus, Denmark

^2^Institute of Clinical Medicine, Aarhus University, Aarhus, Denmark

^3^Department of Anesthesiology and Intensive Care, University Hospital of Southwest Jutland Esbjerg, Esbjerg, Denmark.

^4^Department of Paediatrics and Adolescent Medicine, Lillebaelt Hospital, University Hospital of Southern Denmark, Kolding, Denmark; Department of Regional Health Research, University of Southern Denmark, Denmark.

^5^Department of Forensic Medicine, Aarhus University Hospital, Aarhus, Denmark

^6^Division of Image Processing, Leiden University Medical Center, Leiden, Netherlands

**Address for correspondence**

Jacob Nicolaisen, MD

Department of Cardiology

Aarhus University Hospital

Palle Juul-Jensens Boulevard 99

8200 Aarhus N, Denmark

Phone: +45 21452970

Mail: jacnic@rm.dk

Twitter handle: @JacobNicolaise1

**Supplemental material**

**Expanded materials and methods**

**Experimental protocol**

The day before each catheterization procedure, pigs were fasted overnight with access to drinking water ad libitum. On the day of each procedure, the pigs were pre-medicated with a weight-adjusted mixture of 0.5 mg/kg of midazolam (Accord Healthcare, Gothenburg, Sweden) and 4 mg/kg of azaperone (Janssen Animal Health, Beerse, Belgium) before anesthesia induction using 0.5 mg/kg of intravenous etomidate (Hypnomidate, Janssen-Cilag, UK). Following endotracheal intubation, the pigs were mechanically ventilated with 60% oxygen in pressure regulated volume-controlled mode (S/5 Avance, Datex-Ohmeda, GE Healthcare, UK). Anesthesia was maintained with Sevoflurane (Abbvie A/S, Denmark), to a mean alveolar concentration (MAC) of 1.2 to 1.4%, together with continuous intravenous infusion of fentanyl 0.02 mg/kg/hr (Hameln Pharmaceuticals, Germany). During the procedure, continuous monitoring of electrocardiogram, oxygen saturations, end-tidal CO2 and arterial blood pressure were performed.

The pigs received 1g of intravenous ampicillin (PharmaCoDane, Herlev, Denmark) before and after the catheterization procedures. To minimize peri-procedural arrhythmia, the animals were pre-treated with 25 mg atenolol (Takeda Pharma) once daily and 200 mg amiodarone (Sandoz, Copenhagen, Denmark) twice daily for one week pre- and post-procedure.

**Optical coherence tomography acquisition and analysis**

Pre-stent OCT acquisitions were performed with either optical frequency domain imaging (OFDI, Terumo Medical Corporation, Tokyo, Japan) or the Ilumien C7 OCT system (St. Jude Medical, MN, USA). The BRS size was based on OCT measurements and was implanted in one or two main coronary arteries. Following BRS deployment, the instrumented arteries were evaluated by OCT. In case of severe malapposition detected by OCT, post-dilatation was performed followed by a final OCT acquisition.

**OCT recordings were analyzed in QCU-CMS (Leiden University, Netherlands). Baseline and follow-up OCT scans were matched on frame level by identifiable landmarks including side branches, stent edges and calcified plaques. OCT acquisitions were analyzed with a sample rate of 1 mm.**

**The adjustable strut analysis in QCU-CMS was used for the Desolve and Absorb BRS. The reflective Magmaris stent struts inhibited visualization of the abluminal boarder, hence analysis with fixed strut thickness was utilized in the Magmaris group (supplementary figure 1). OCT analysis was performed in the following 4 steps. 1) The lumen contour was traced at the abluminal side of stent struts at baseline and on the neointimal tissue at follow-up. 2) Stent strut delineation. 3) The stent contour was automatically detected with a pre-specified thickness of 157** $\mu m$ **for Absorb BRS and 150** $\mu m$ **for DESolve and Magmaris BRS. Manual corrections were performed if needed. 4) Stent struts were individually characterized as malapposed, jailing, tissue covered or not tissue covered.**

**OCT endpoints included in-stent minimal and mean lumen area at 6-months follow-up, mean stent area, minimal stent area, mean neointimal hyperplasia area,** mean neointimal thickness, mean extra-stent lumen area, strut malapposition and strut coverage.

**Malapposition was defined as a visual separation of the strut box and the lumen contour. A strut was defined as covered if a single layer of tissue was visible on the luminal side.**

**Qualitative OCT assessment of the atherosclerosis was performed using the standard plaque classifications. A lipid plaque, or fibrous cap atheroma, was defined as a signal-poor region with poorly delineated boarders and low reflection below a fibrous cap. The fibrous cap may contain highly reflective foamy macrophages. Neoatherosclerosis was defined as highly reflective foamy macrophage accumulation.**

**Supplemental tables**

**Supplementary table 1: Additional baseline and follow-up characteristics**

| Stented vessels  (n=19) | Magmaris  (n=7) | Absorb  (n=6) | Desolve  (n=6) |
| --- | --- | --- | --- |
| Reference diameter (mm) *  Baseline:  Follow-up: | 3.30 ± 0.43  3.16 ± 0.65 | 3.00 ± 0.65  2.96 ± 0.70 | 3.27 ± 0.35  2.90 ± 0.38 |
| Reference area ($\mathbf{m}\mathbf{m}^{\mathbf{2}}\boldsymbol{)}$ *  Baseline:  Follow-up: | 8.67 ± 2.19  8.40 ± 3.29 | 7.46 ± 3.22  7.30 ± 3.26 | 8.55 ± 1.77  6.75 ± 1.71 |
| Treated vessels (n)  LAD  LCx  RCA | 2  1  4 | 2  1  3 | 1  1  4 |
| Nominal stent length (mm) | 20.7 ± 4.50 | 18.0 ± 6.32 | 20.0 ± 6.45 |
| Nominal stent diameter (mm)  2.5  3.0  3.5 | 0  4  3 | 0  4  2 | 2  3  1 |

Supplementary table 1: Results are presented as mean ± SD or counts. * measured by OCT.

**Supplementary table 2: Additional matched baseline and 6-months OCT results**

| OCT endpoints  (n=19 stented vessels) | Magmaris  (n=7) | Absorb  (n=6) | Desolve  (n=6) | P-value |
| --- | --- | --- | --- | --- |
| Mean neointimal thickness (mm)  Baseline:  Follow-up: | -  0.27 ± 0.08 | -  0.28 ± 0.11 | -  0.41 ± 0.15 | -  0.10 |
| Extra stent lumen area ($\boldsymbol{m}\boldsymbol{m}^{\boldsymbol{2}}\boldsymbol{)}$  Baseline:  Follow-up: | 0.017 ± 0.01  - | 0.17 ± 0.14  - | 0.23 ± 0.19  - | 0.002  - |
| Covered struts (%) * † | 100 [100;100] | 100 [100;100] | 100 [100;100] | - |

Supplementary table 2: Results are reported as mean ± SD. *Median [IQR]. †Analyzed on strut level.

**Supplemental figures**

**Supplementary figure 1: Matched OCT analysis**


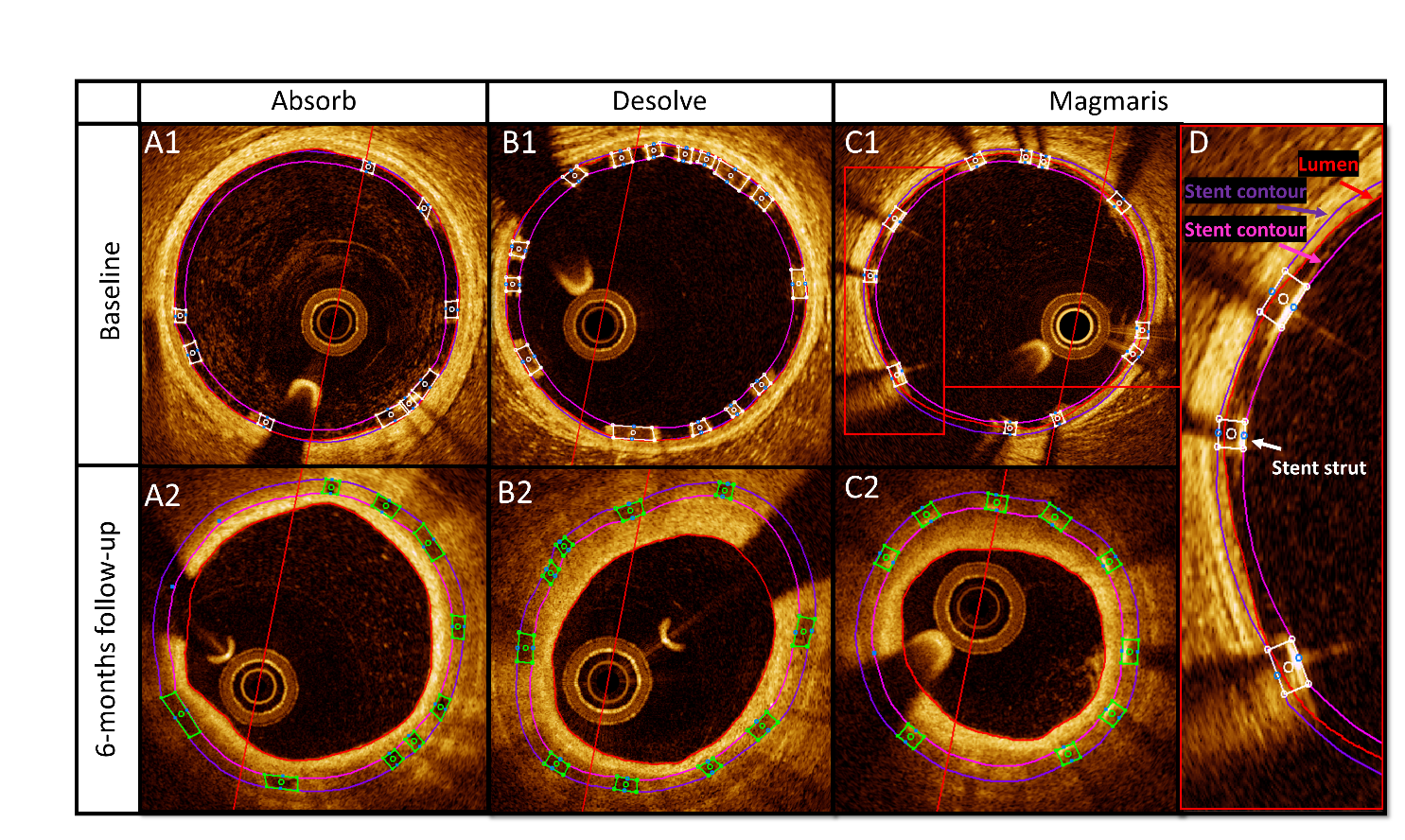


Supplementary figure 1: Matched OCT analysis of Absorb (A1+A2), Desolve (B1+B2) and Magmaris BRS (C1+C2+D). A1+B1+C1) Cross-sections showing the baseline analysis of the Absorb, Desolve and Magmaris OCT analysis. A2+B2+C2) illustrates the 6-months follow-up analysis of the Absorb, Desolve and Magmaris BRS. D) Close-up of the baseline Magmaris OCT analysis depicting the lumen contour (red line), the abluminal (purple line) and luminal (pink line) stent contours and stent struts (white boxes).

**Supplementary figure 2: Changes in lumen area from baseline to 6-months follow-up**


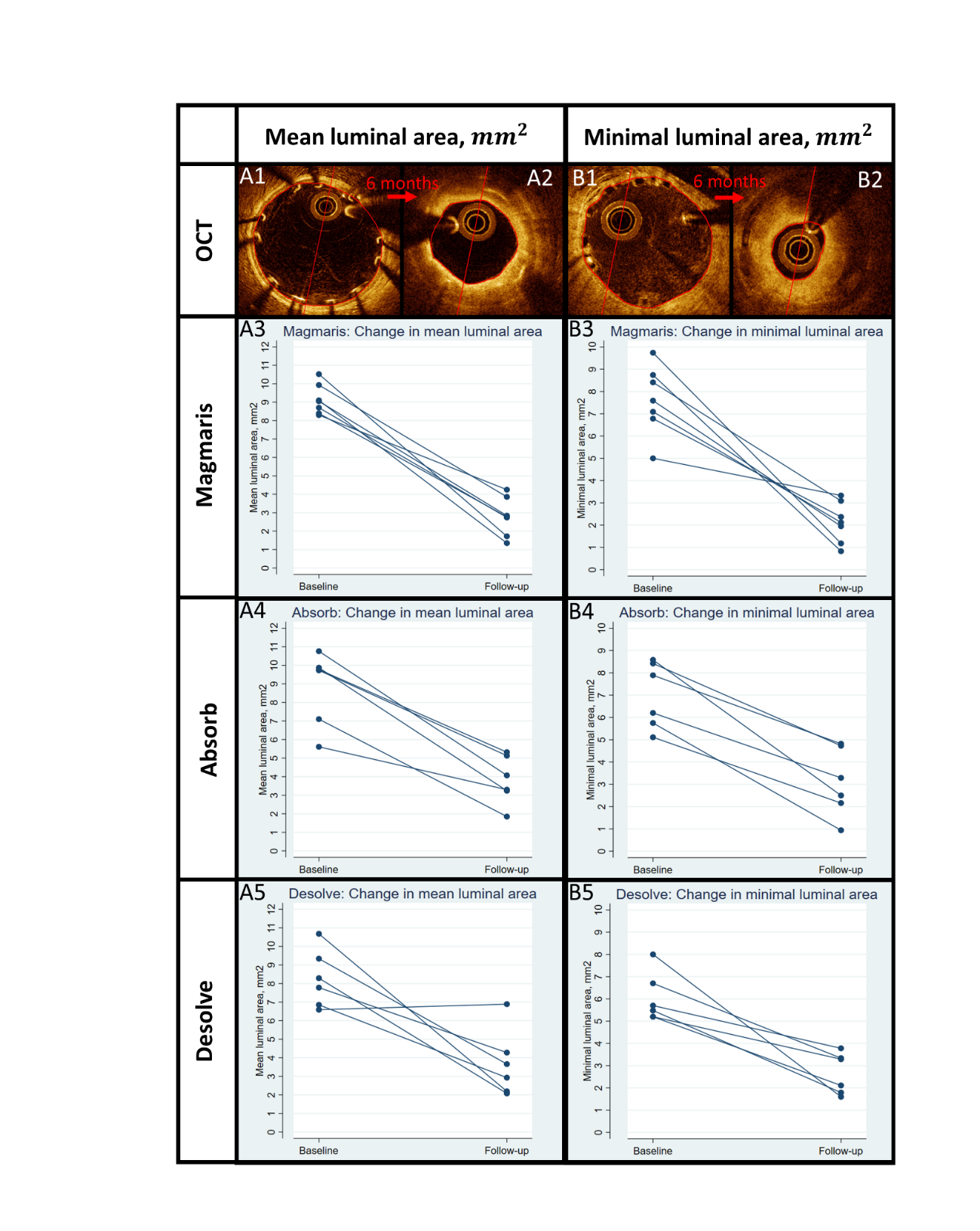


Supplementary figure 2: Changes in mean lumen area (A1-A5) and minimal lumen area (B1-B5) from baseline to 6-months follow-up. A1+A2: Matched OCT cross-section from a Magmaris BRS implantation with traced lumen contour (red) from baseline (A1) to follow-up (A2). B1+B2: Matched OCT cross-sections from an Absorb BRS implantation with traced lumen contour at baseline (B1) and follow-up (B2). The changes in mean and minimal lumen area from baseline to follow-up are depicted for the Magmaris BRS (A3+B3), the Absorb BRS (A4+B4) and Desolve BRS (A5+B5).

**Supplementary figure 3: Changes in stent area from baseline to 6-months follow-up**


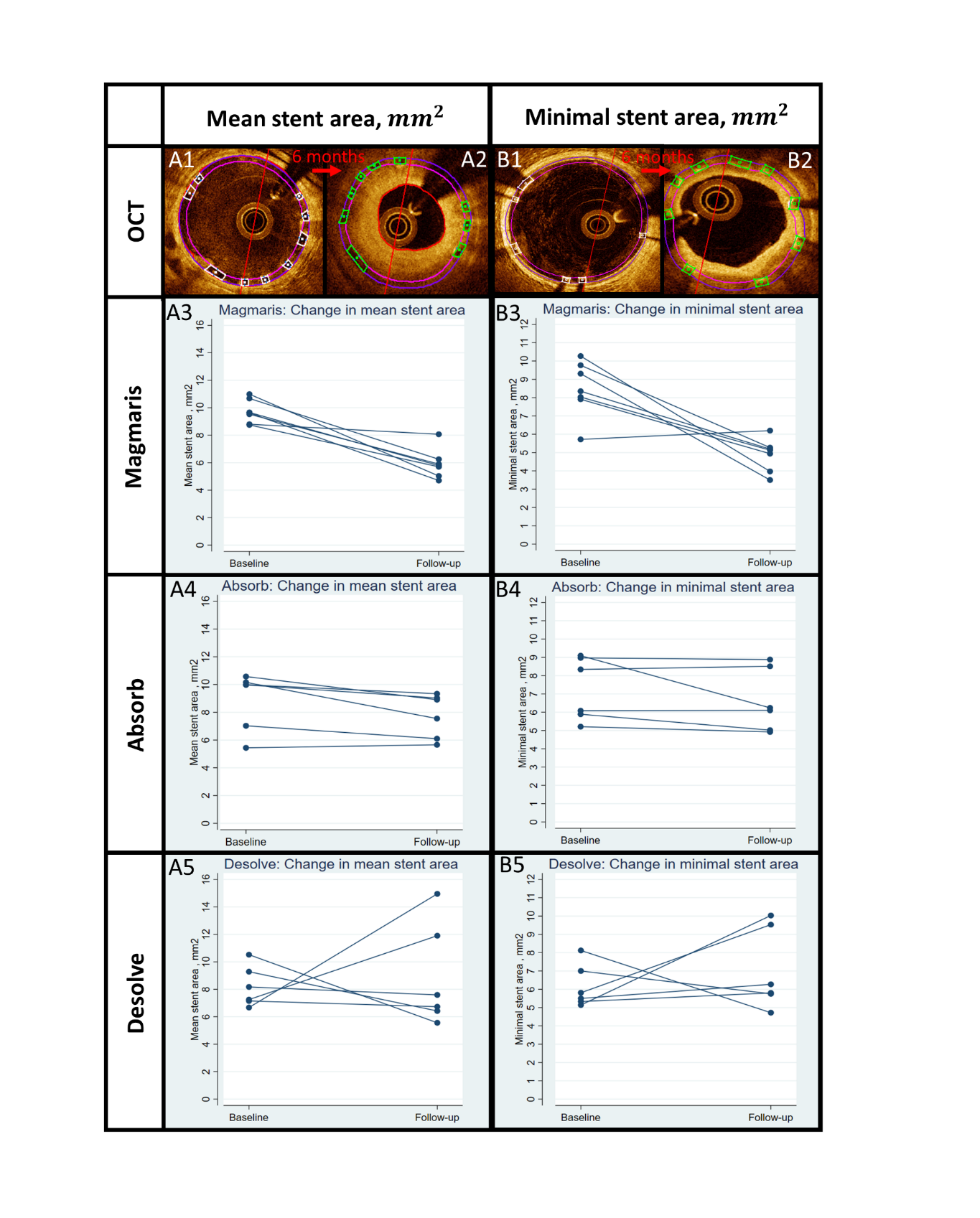


Supplementary figure 3: Changes in mean stent area (A1-A5) and minimal stent area (B1-B5). Matched OCT cross sections illustrating Magmaris BRS loss in stent area from baseline (B1) to follow-up (B2). A1+A2: Matched Cross-sections showing a stable stent area from baseline (A1) to follow-up (A2) despite loss in lumen area in an Absorb BRS implantation. Graphs illustrating the change in mean stent area (A3-A5) and minimal stent area (B3-B5) from baseline to 6-month follow-up for Magmaris, Absorb and Desolve BRS.

**Supplementary figure 4: Serial changes in luminal area from baseline to 12-months follow-up**


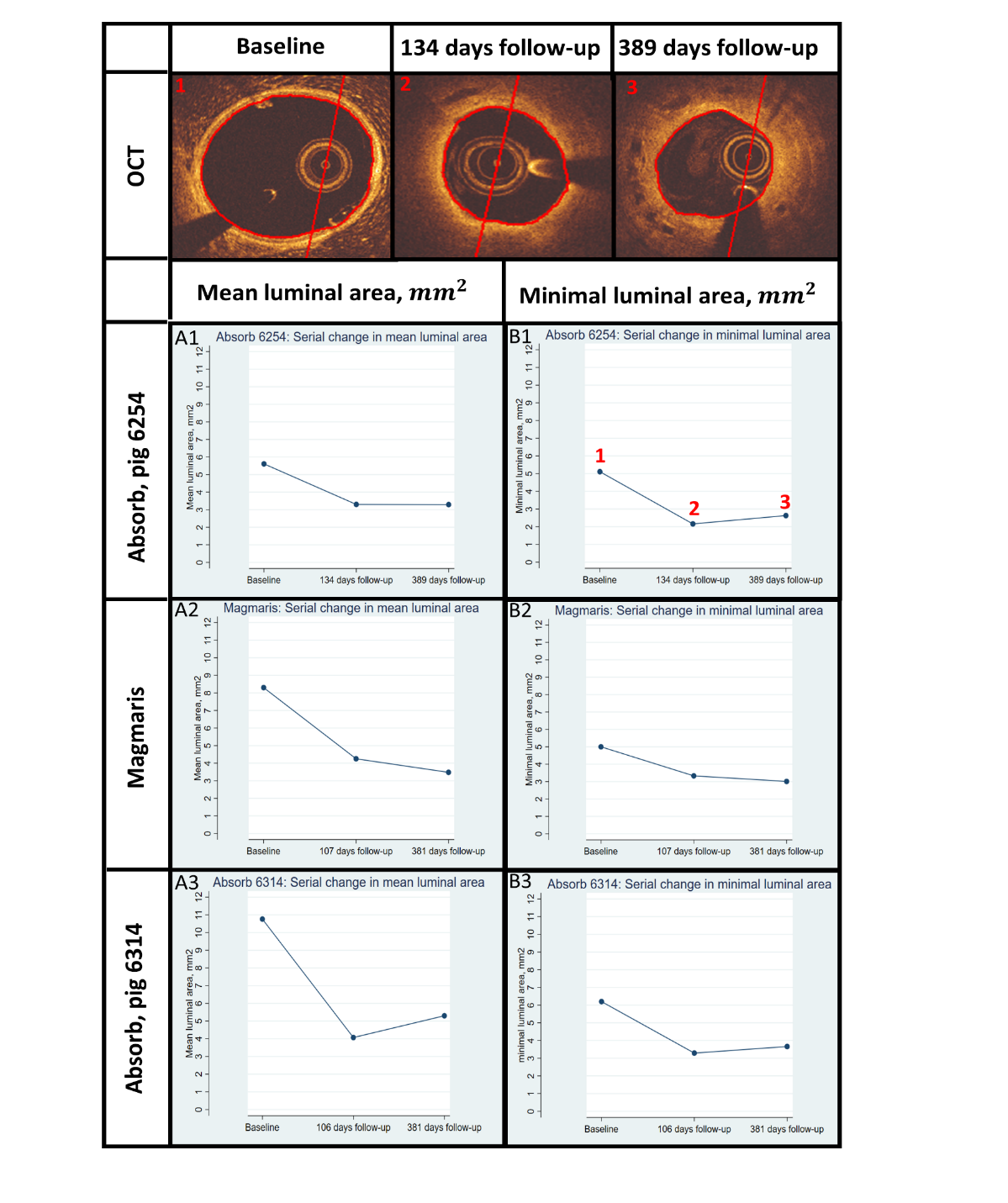


Supplementary figure 4: Serial changes in lumen area from baseline, 3-4 months follow-up and 1-year follow-up. Serial changes in mean lumen area (A1-A3) and minimal lumen area (B1-B3). A1: Serial change in mean luminal area for pig 6254 from baseline, 134 days follow-up and 389 days follow-up. B1: Serial change in minimal luminal area for pig 6254 from baseline (1), 134 days follow-up (2) and 389 days follow-up (3). 1+2+3: OCT cross-sections of minimal luminal area for pig 6254 at baseline, 134 days follow-up and 389 days follow-up. A2+B2: Mean and minimal luminal area for a Magmaris BRS implanted pig. A3+B3: Mean and luminal area for Absorb BRS implanted pig 6314.
